# Super-hydrophobic Silver-Doped TiO_2_ @ Polycarbonate Coatings Created on Various Material Substrates with Visible-Light Photocatalysis for Self-Cleaning Contaminant Degradation

**DOI:** 10.1038/srep42932

**Published:** 2017-02-20

**Authors:** Zhengjian Li, Zongzhao Sun, Zhiqiang Duan, Rui Li, Yanli Yang, Jingyi Wang, Xiaoxia Lv, Wei Qi, Hua Wang

**Affiliations:** 1 Institute of Medicine and Material Applied Technologies, College of Chemistry and Chemical Engineering, Qufu Normal University, Qufu, 273165, P. R. China

## Abstract

In the present work, a facile and efficient fabrication method has been developed for creating super-hydrophobic coatings of silver-doped TiO_2_@polycarbonate (TiO_2_ (Ag)@PC) on the substrates of different materials with photocatalytic self-cleaning performances simply by the “dipping and drying” process. The substrates were first patterned with glue and then deposited with the dopamine-capped TiO_2_ (Ag)@PC (DA-TiO_2_ (Ag)@PC) nanocomposites, followed by the further etching with dimethylbenzene. The so prepared super-hydrophobic E-DA-TiO_2_(Ag)@PC coatings could present the lotus leaf-like porous architectures, high adhesion stability, and especially the visible-light photocatalysis for organic contaminant degradation, thus promising the wide outdoor and indoor applications like water proofing, metal erosion protection, and surface self-cleaning.

The surface super-hydrophobicity phenomenon, with water contact angles (CAs) higher than 150°, of some natural plants such as lotus leaf termed the “lotus effect”^1^ has attracted increasing attentions. It is well established that the surface of a lotus leaf consists of numerous micrometer-sized papillae of wax crystals with smaller nanometer-sized protrusions showing the super-hydrophobicity^1^. In recent years, the super-hydrophobic materials or lotus leaf-like surfaces have been used in many fields such as the waterproofing, anti-fouling, self-cleaning, fluid drag reduction, and antibacterial^2^,^3^. Traditionally, the super-hydrophobic coatings were generally constructed by the complicated procedures comprising of rough surfaces covered with some low surface energy modifiers^4^. Up to date, numerous techniques have been reported for creating super-hydrophobic surfaces most known as the etching^5^,^6^,^7^, deposition^8^,^9^,^10^, assembling^11^,^12^,^13^, and phase separation^14^,^15^. Moreover, some materials of low surface energy like long-chain alkanes, fluorosilane, and functional polymers were extensively employed to fabricate the super-hydrophobic surfaces^4^,^14^,^15^,^16^,^17^,^18^. However, these fabrication techniques may generally involve the harsh preparation conditions, complex steps, and expensive chemical modifiers. Alternatively, the phase separation technique, which can fabricate straightforward super-hydrophobic surfaces with different polymers^14^,^19^,^20^,^21^, has been proven to be a facile, fast, and effective way in producing the controllably-rough super-hydrophobic surfaces without any low energy modifiers. For example, Xinhong Li and co-workers^19^ described the fabrication of super-hydrophobic films using poly (vinyl chloride) together with the control of the ethanol-to-water ratios, showing a porous structure and a water CAs of 160°. Zhang *et al*. prepared the super-hydrophobic coatings on various material substrates using polycarbonate (PC)^14^. Also, the super-hydrophobic surfaces were constructed by Motoshi Yamanaka *et al*. using perfluoroalkyl chain-containing organogelators^22^. However, most of the pioneering super-hydrophobic films or coatings on various substrates by the phase separation method can be generally challenged either by the poor adherent stability on the substrates or the formidable organic contaminations on the coating surfaces. For example, organic contaminants may be absorbed on the super-hydrophobic surfaces so tightly to be removed as time goes on.

Recent years have witnessed the wide applications of some photocatalysts like titanium dioxide (TiO_2_) for the photocatalytic degradation of organic contaminations like dyes^23^,^24^. TiO_2_ with some unique properties such as high stability, high reactivity, low toxicity, and cost-effectiveness is thought to be an ideal component for building the photocatalysic nanomaterials or films^21^,^25^. However, the poor solar absorbance of TiO_2_ materials can bring about the considerably-low efficiency of photocatalysis under visible light^23^. As an efficient solution to this problem, doping TiO_2_ with some noble metal elements can increase the utilization of visible light toward the improved photocatalysis^26^. For example, silver (Ag)-doped TiO_2_ (TiO_2_ (Ag)) was applied to attain the increased photocatalytic degradation ability^27^,^28^ for some dyes like rhodamine B (RhB), methylene blue (MB), and methyl orange (MO)^29^,^30^.

In the present work, a facile and efficient fabrication technique has been proposed for the first time for creating highly stable super-hydrophobic coatings on various material substrates with photocatalytic self-cleaning performances simply by a “dipping and drying” process (Fig. 1). Through following the phase separation way, herein, PC was employed as a readily-available polymer model matrix to embed dopamine (DA)-encapsulated silver-doped TiO_2_ (DA-TiO_2_(Ag)), yielding the super-hydrophobic E-DA-TiO_2_(Ag)@PC coatings on the various material substrates which surfaces were pre-patterned with glue. The so developed super-hydrophobic coatings were demonstrated with lotus leaf-like porous architectures, high substrate adhesion stability, visible-light photocatalytic contaminant degradation, and self-cleaning performances for the wide practical applications.

## Results

### Preparation and characterization of E-DA-TiO_2_ (Ag)@PC coatings

**Figure 1:**
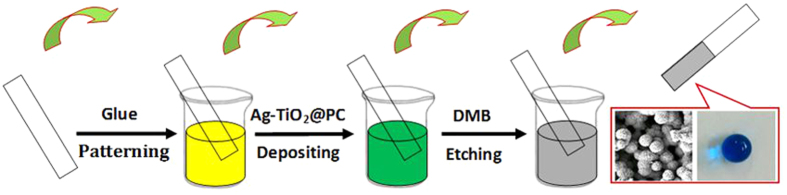
Schematic illustration for the phase separation-based fabrication procedure of super-hydrophobic photocatalytic E-DA-TiO_2_ (Ag)@PC coatings deposited on the glue-patterned substrates of different materials by the “dipping and drying” procedure, including glue patterning, DA-TiO_2_ (Ag)@PC deposition, and DMB etching, leading to the formation of the coatings showing the lotus-like porous morphology and super-hydrophobicity.

The super-hydrophobic coatings were created on various substrates based on the phase separation procedure simply by using a “dipping and drying” process at room temperature, as schematically illustrated in Fig. 1. A coating formula consisting of DA, silver-doped TiO_2_ (DA-TiO_2_ (Ag)), and PC was prepared and defined as the DA-TiO_2_ (Ag)@PC composite, in which DA was employed to help the doping of silver into TiO_2_ matrix by reduction and increase the visible-light absorption of TiO_2_ toward the improved photocatalysis, in addition to the encapsulation of TiO_2_ (Ag) particles into PC matrix as mentioned afterwards. Here, the substrates of different materials were first patterned with glue, and then dipped into the DA-TiO_2_ (Ag)@PC suspension to deposit the composite films by the phase separation route^14^,^15^. Furthermore, dimethylbenzene (DMB) was employed as the non-solvent to etch the DA-TiO_2_ (Ag)@PC films, resulting in the E-DA-TiO_2_ (Ag)@PC coatings with enhanced surface roughness to access the super-hydrophobicity^14^,^17^. Importantly, high adhesive ability of coatings could thus be expected on these substrates.

**Figure 2:**
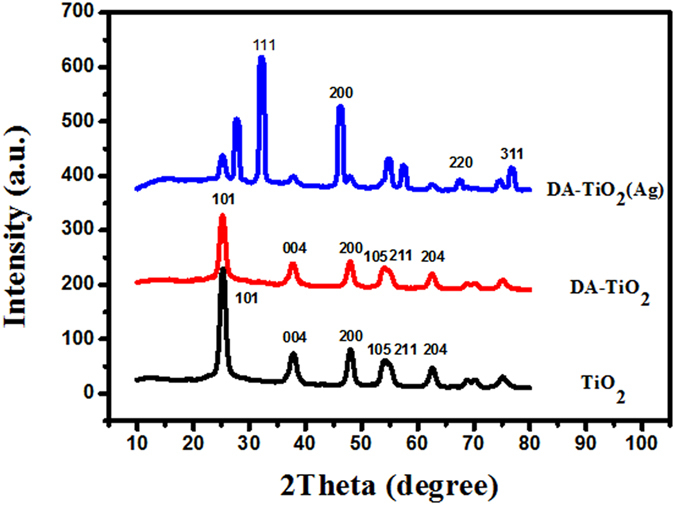
XRD patterns of TiO_2_, DA-TiO_2_, and DA-TiO_2_ (Ag) composites, where the diffraction peaks of TiO_2_ for TiO_2_ and DA-TiO_2_, and those of Ag^0^ for DA-TiO_2_ (Ag) composites are indicated.

**Figure 3:**
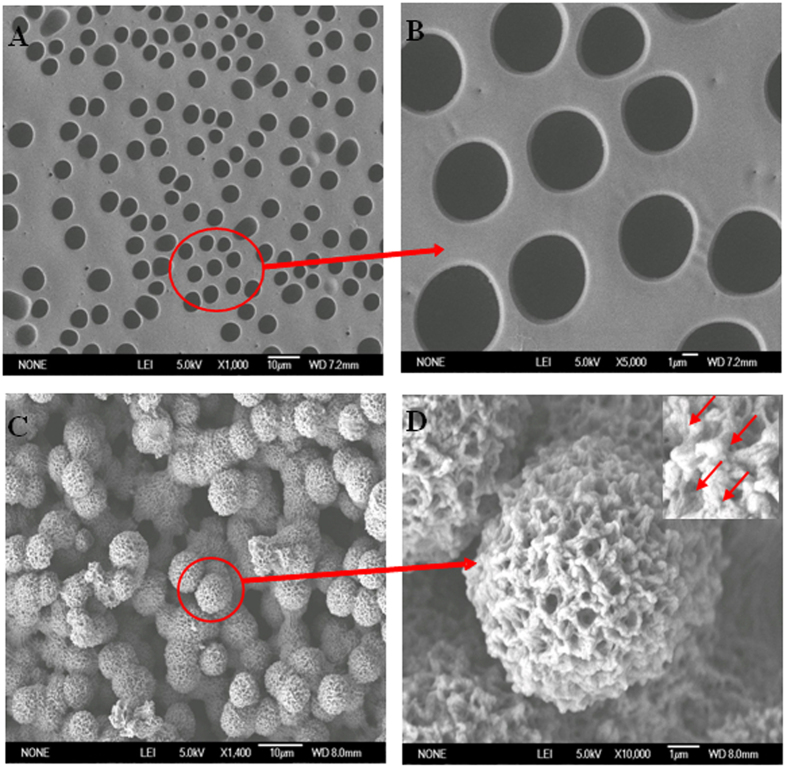
SEM images of the surface morphologies of (**A**) DA-TiO_2_ (Ag)@PC coatings and (**B**) its magnitude-amplified view, (**C**) E-DA-TiO_2_ (Ag)@PC coatings and (**D**) its magnitude-amplified view (insert: the amplified part with DA-TiO_2_ (Ag) composites indicated by red arrows).

X-ray diffraction (XRD) analysis of TiO_2_, DA-TiO_2_, and DA-TiO_2_ (Ag) composites were first conducted, with the data shown in Fig. 2. One can note that these products can possess the diffraction peaks of TiO_2_ at the same 2 Theta positions of anatase type, including 25.7° (101), 37.9° (004), 47.9° (200), 53.7° (105), 55.0° (211), and 62.9° (204). By comparison, DA-TiO_2_ (Ag) composites display additionally the characteristic diffraction peaks of silver such as 32.2° (111), 45.5° (200), 67.5° (220), and 76.2° (311), which are basically consistent to those of Ag^0^ doped in the composites. Moreover, scanning electron microscopy (SEM) imaging was comparably conducted to characterize the morphological structure of the E-DA-TiO_2_ (Ag)@PC coatings (Fig. 3). One can observe that before the DMB etching the coatings exhibited a dense honeycombed structure with limited micro-sized spherical pores (Fig. 3A and B). As shown in Fig. 3C, the yielded DA-TiO_2_ (Ag)@PC coatings by the DMB etching could manifest the uniformly-defined spheres or papillae stacked with lotus leaf-like porous architectures with an average particle size of about 6.0 μm, as revealed more clearly in the magnitude-amplified view (Fig. 3D) showing the encapsulated DA-TiO_2_ (Ag) composites that were indicated by the red arrows (insert). Accordingly, the surfaces of E-DA-TiO_2_ (Ag)@PC coatings could feature the micro-nano-binary roughness structures like the lotus leaves, where air might be trapped in between the cavities of the porous papillae so as to repel the water droplets, which may essentially explain the super-hydrophobicity of the coatings in terms of CAs over 150°^14^,^18^.

### Adhesion stability of E-DA-TiO_2_ (Ag)@PC coatings on substrates

**Figure 4:**
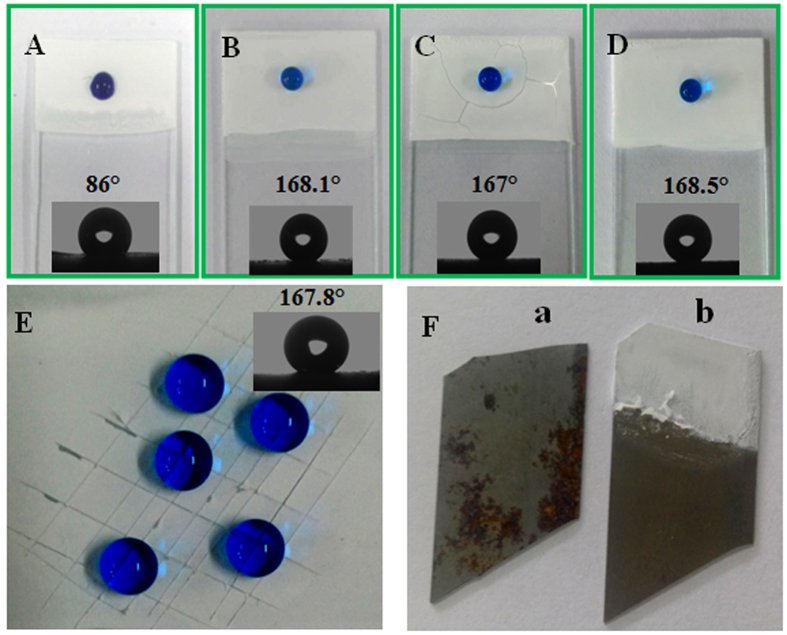
The performance comparison among the DA-TiO_2_ (Ag)@PC coatings on the (**A**,**B**) glue-patterned or (**C**) free substrates in comparison to (**D**) the pure PC coatings on the glue-patterned substrate, where the DA-TiO_2_ (Ag)@PC coatings were constructed on the glue-patterned substrates (**A**) before and (**B**) after the DMB etching. All of the surface profiles were checked with water droplets after being exposed under xenon lamp for 2 h. (**E**) Scratch tests for the E-DA-TiO_2_ (Ag)@PC coatings created on the glass slides, of which the hdrophobic performances were checked using water droplets after the knife scratches. (**F**) The corrosion-against tests for the iron blocks shelled (a) without and (b) with E-DA-TiO_2_ (Ag)@PC coatings (the lower part with the coatings peeled off after the tests), where the tests were conducted by separately immersing the iron blocks in the saturated NaCl solutions over night.

The adhesion stability of E-DA-TiO_2_ (Ag)@PC coatings on the substrates was evaluated including the hydrophobicity-endowed corrosion resistance for metal substrates. One can note that the DA-TiO_2_ (Ag)@PC coatings could display the different hydrophobicities in terms of contact angles before (Fig. 4A) and after (Fig. 4B) the DMB etching. Moreover, the developed nanocomposite coatings could exhibit much higher stability on the glue-patterned substrates (Fig. 4B) than the bare ones (Fig. 4C). Despite no obvious change was noted in the surface profiles of water droplets, herein, the apparent crazing could be witnessed for the coating on the bare substrate once exposed under xenon lamp for 2 h, together with the slightly decreased hydrophobicity in terms of CAs (Fig. 4C). It confirms that the glue patterning for substrates could dramatically enhance the substrate-adhesive ability of super-hydrophobic coatings due to the used glue can generate the strong bonds with a variety of substrates^31^. Moreover, compared to the pure PC coatings (Fig. 4D), the so prepared E-DA-TiO_2_ (Ag)@PC coatings could show no significant change in the hydrophobicity (Fig. 4B). Yet, they could possess the additional functions of photocatalytic dye degradation and self-cleaning, mainly resulting from the inclusion of DA-TiO_2_ (Ag) as demonstrated afterwards. Furthermore, the mechanical robustness was examined by the knife scratch tests for the E-DA-TiO_2_ (Ag)@PC coatings created on glass slides (Fig. 4E). One can note that the water droplets on the scratched surface of coatings could well retain the profiles of the high hydrophobicity with the average CA of about 167.8°, showing the pretty high substrate adhesion and mechanical robustness against the abrading. Additionally, the adhesion of the E-DA-TiO_2_(Ag)@PC coatings on steel substrate was evaluated by the Cross-cut tape test of ASTM D3359B-02 Method B^32^, showing the adhesion rank 4 (good) accordingly.

Moreover, the unique super-hydrophobic performances of the E-DA-TiO_2_ (Ag)@PC coatings were applied in the corrosion resistance for iron plates as the metal example. The composite coatings were shelled on the glue-patterned iron plates to be immersed into the saturated NaCl solutions overnight, taking the bare one for the control comparison (Fig. 4F). Expectedly, the resulted iron plates could be well prevented from the rusting due to the screening of super-hydrophobic coatings (seeing the lower part exposed with the coatings peeled off).

### E-DA-TiO_2_ (Ag)@PC coatings on the substrates of various materials

**Figure 5:**
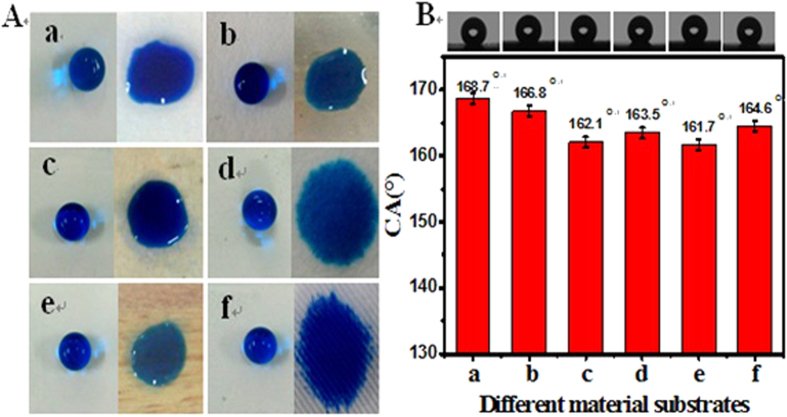
(**A**) Hydrophobic comparison of surface profiles with the water droplets of MB among the E-DA-TiO_2_ (Ag)@PC coatings (left lane) on the substrates of different materials of (a) glass, (b) iron, (c) ceramics, (d) paper, (e) wood, and (f) textile, taking the uncoated ones (right lane) as the controls; (**B**) the corresponding surface hydrophobicities (CAs) of the E-DA-TiO_2_ (Ag)@PC coatings on the substrates of different materials indicated. (Insert: the photographs of corresponding water droplets for measuring CA.

The super-hydrophobic E-DA-TiO_2_ (Ag)@PC coatings were textured separately on the substrates of various materials including glass, iron, ceramics, paper, wood, and textile (Fig. 5A, left). Obviously, the nanocomposite coatings could visually display the largely improved surface hydrophobicity shown by the surface profiles of water droplets, as compared to the bare substrates of corresponding materials (Fig. 5A, right). For example, water droplets would not be absorbed into the textile matrix after being deposited with the hydrophobic coatings (Fig. 5A, f). More importantly, the so created E-DA-TiO_2_ (Ag)@PC coatings on these substrates could illustrate basically the approximate CAs over 160° to attain the surface super-hydrophobicity (Fig. 5B), indicating that the developed coatings are generally applicable for the substrates of different kinds of materials.

### Super-hydrophobic durability of the E-DA-TiO_2_ (Ag)@PC coatings

**Figure 6:**
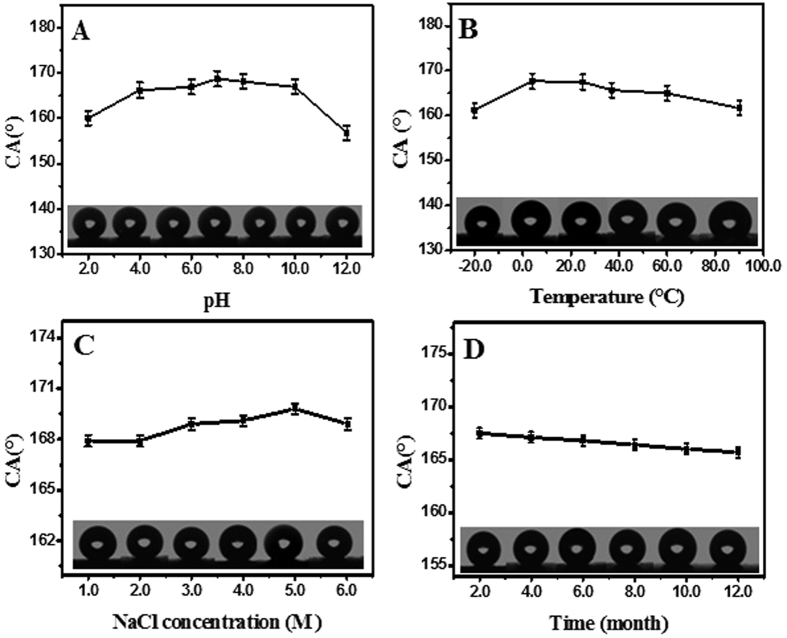
The hydrophobic performances (CAs) of the E-DA-TiO_2_ (Ag)@PC coatings depending on the varying testing conditions of (**A**) pH values, (**B**) temperature, (**C**) ion strengths (NaCl concentrations) and (**D**) storage time, where the CAs were recorded for the E-DA-TiO_2_ (Ag)@PC coatings created on the glue-patterned glass substrates and further immersed overnight into the testing solutions, expect for the ones with the storage time for one year (insert: the photographs of corresponding water droplets for measuring CAs).

The environmental durability of the E-DA-TiO_2_ (Ag)@PC coatings was investigated under different conditions, including pH values, temperature, ion strengths, and stable time (Fig. 6). It was discovered that the E-DA-TiO_2_ (Ag)@PC coatings tested could retain the wonderful surface super-hydrophobic characteristics in water CAs in the range of pHs from 2.0 to 12.0 (Fig. 6A), temperature from −20 °C to 90 °C (Fig. 6B), NaCl concentration up to 6.0 M (Fig. 6C), and the stable test time over one year (Fig. 6D). The data above validate that the super-hydrophobic E-DA-TiO_2_ (Ag)@PC coatings on the glue-patterned substrates could display the desirably high environmental durability, mainly resulting from the synergic combination of stable super-hydrophobic coatings with the strongly adhesive glue patterns^31^, thus promising the practical applications under a variety of conditions.

### Photocatalytic dye degradation performances of the E-DA-TiO_2_ (Ag)@PC coatings

**Figure 7:**
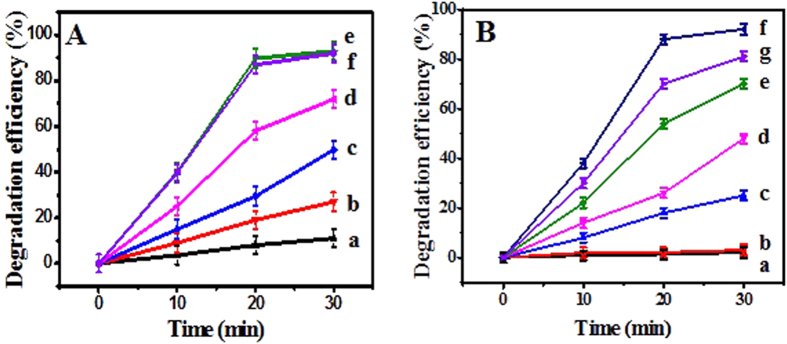
The photocatalytic degradation efficiencies (%) of RhB on E-DA-TiO_2_ (Ag)@PC coatings depending on (**A**) the Ag dosages in TiO_2_ (a-f refers to 0, 1.0, 2.0, 3.0, 4.0 and 5.0 mM), and (**B**) the DA-TiO_2_ (Ag) concentrations (a-g refers to 0, 0.10, 0.20, 0.30, 0.40, 0.50, and 0.60 wt%) in PC (10 wt%), where the photocatalytic degradation tests were conducted under visible light of xenon lamp.

**Figure 8:**
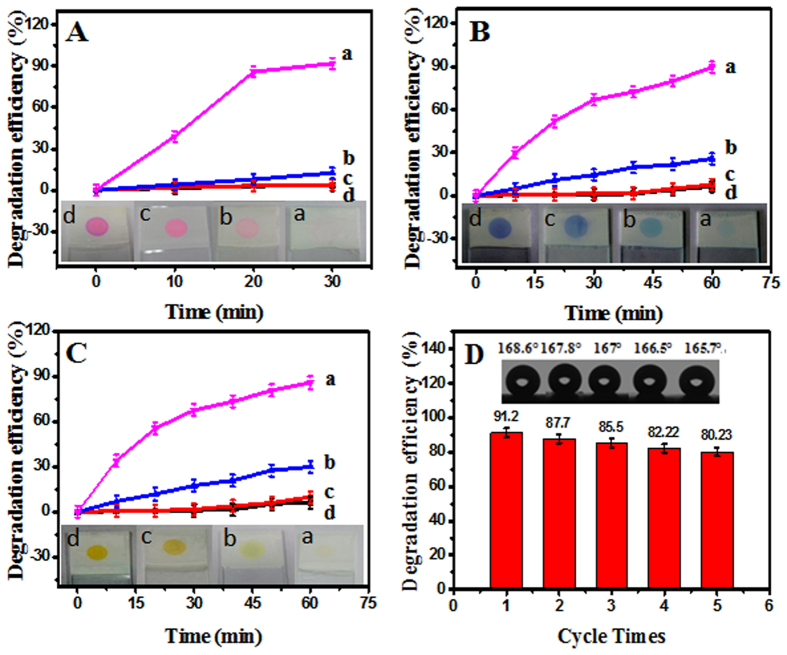
Photocatalytic degradation efficiencies for the dye droplets of (**A**) RhB, (**B**) MB and (**C**) MO deposited separately on (c) PC coatings, (b) TiO_2_ @ PC coatings, and (a) E-DA-TiO_2_ (Ag)@PC coatings under visible light for 30 min (RhB) or1 h (MB and MO), taking (d) the ones in dark as the controls (Insert: the photographs of the dye droplets degraded on the corresponding coatings). (**D**) The cycling times-depending degradation efficiencies for RhB droplets and hydrophobic performances (CAs) of E-DA-TiO_2_ (Ag)@PC coatings (insert: the photographs of corresponding water droplets for measuring CAs).

It is well established that TiO_2_ materials could possess the substantially low photocatalysis under visible light, which may be generally improved by doping some noble metals like Ag element^27^,^28^.In the present work, the photocatalytic performances of the E-DA-TiO_2_ (Ag)@PC coatings were investigated for the dye degradation on RhB on the coatings under visible light (Fig. 7). Herein, the photocatalytic dye degradation capabilities of the composite coatings were determined separately depending on the amounts of Ag and the DA-TiO_2_ (Ag). The photocatalytic degradation efficiencies were obtained by the equation of (A_0_-A)/A_0_ × 100%, where A_0_ and A refer to the absorbance values of dye droplets before and after the photocatalytic degradation, respectively. As shown in Fig. 7A, the photocatalytic activities of the E-DA-TiO_2_ (Ag)@PC coatings significantly increased with the increase in the Ag dosages up to 5.0 mM, showing the maximized photocatalytic efficiency. Meantime, the use of DA-TiO_2_ (Ag) of 0.50 wt% in PC could attain the highest photocatalytic degradation efficiencies (Fig. 7B). Moreover, a comparison of photocatalytic degradation performances was conducted among the composition-various coatings of E-DA-TiO_2_ (Ag)@PC, TiO_2_@PC, and PC, taking the ones in dark as the controls (Fig. 8). Here, the dye degradation efficiencies of these coatings were systematically evaluated using three organic dyes of neutral RhB (Fig. 8A), positively charged MB (Fig. 8B), and negatively charged MO (Fig. 8C), which droplets were separately placed on the coatings for different time under the visible light. One can find that three dye droplets on E-DA-TiO_2_ (Ag)@PC coatings could display the highest degradation efficiencies (over 90%) under the visible light (curve **a**), as also shown in the photographs of these dye droplets (insert). By comparison, the TiO_2_@PC coatings could exhibit a little of photocatalytic degradation (curve **b**), but their degradation efficiencies for three dye droplets (below 30%) were much lower under the visible light presumably due to the absence of Ag element. Moreover, no dye degradation occurred obviously on the PC coatings without the photocatalysts of TiO_2_ or TiO_2_ (Ag) (curve **c**), so did the ones on the E-DA-TiO_2_ (Ag)@PC coatings in dark (curve **d**). These data indicate that the so prepared E-DA-TiO_2_ (Ag)@PC coating could display the considerably high photocatalytic degradation for the organic dyes under visible light, with the exposure time within 30 min for RhB and 1 h for MB or MO. Herein, the introduction of Ag additive can, on the one hand, enhance the harvest of the visible light of TiO_2_ by improving their surface electronic conductivity and plasmon resonance photosensitization. On the other hand, it can endow the formation of Schottky barrier between Ag and TiO_2_ semiconductor may contribute to the separation of electro−hole pairs towards the improved photocatalysis under visible light^27^,^28^. Additionally, the use of DA as an enediol ligand could additionally facilitate the tunable photocatalysis responses of TiO_2_ in the visible spectral region by adjusting the coordination geometry of Ti atoms on the TiO_2_ surface^33^,^34^. In particular, the high viscosity of DA can also endow the TiO_2_ (Ag) composites firmly embedded into the PC matrix. The environmental stability and application cycles of the E-DA-TiO_2_ (Ag)@PC coatings were explored (Fig. 8D). It was discovered that the developed coatings could be recyclably applied for five times without a significant change in the super-hydrophobic performances and dye degradation abilities, resulting from the employment of strongly viscous DA and glue that were the substrates, respectively. Therefore, the developed fabrication strategy can be expected to construct the coatings on the substrates of various materials with the desirable super-hydrophobicity and photocatalytic degradation under visible light to achieve the self-cleaning performances.

In summary, the super-hydrophobic coatings have been successfully constructed desirably with lotus leaf-like porous architectures on the substrates of various materials simply by the “dipping and drying” process, showing high super-hydrophobicity, substrate adhesion stability, and visible-light photocatalysis for the self-cleaning contaminant degradation. The developed fabrication route for super-hydrophobic coatings can display some advantages over the traditional methods. First, the super-hydrophobic coatings (CA > 160^o^) were created using the readily available polymer materials (i.e., PC) by the phase separation way, without the need for the modification of low surface energy modifiers and complicated instruments for temperature or humidity control. Second, the Ag-doped TiO_2_ composites could realize the high photocatalysis efficiency for the contaminant degradation under the visible light, in contrast to the common super-hydrophobic surfaces that are readily infected with formidable organic contaminations. Third, the use of DA with considerably-high substrate viscosity can not only help the doping of silver into TiO_2_ matrix by reduction to obtain the strong environmental stability on the substrates, but also increase the visible-light absorption of TiO_2_ (Ag) with further improved photocatalysis. Forth, the introduction of glue to pattern the substrates could dramatically enhance the substrate adhesion of super-hydrophobic coatings with high mechanical robustness against the abrading, making them widely applicable in harsh conditions. Subsequently, this simple “dipping and drying” fabrication procedure can be tailored to mimic the lotus leaf-like micro-nano-binary structure under the mild conditions, especially applicable for various material substrates and complex shapes (i.e., the channels of micro-fluidic devices). Therefore, the developed fabrication strategy offers a facile and efficient way toward the extensive creation of super-hydrophobic and photocatalytic coatings on with lotus leaf-like architectures on various material substrates, thus promising the wide commercial applications for the outdoor equipment (i.e., erosion-against transportation devices) and the indoor furniture (i.e., easily-contaminated kitchen tables). Yet, the other potential functions of the developed versatile coatings like self-cleaning and silver-endowed antimicrobial property should be explored in the future works.

## Methods

### Materials and instruments

Tetrabutyltitanate (TBOT), ethanol (C_2_H_5_OH), acetic acid (CH_3_COOH), polyethylene glycol (PEG, MW 6000), silver nitrate, and dopamine (DA) were purchased from Sinopharm Chemical Reagent Co. (China). Cyanoacrylate glue, polycarbonate (PC), dichloromethane (DCM), dimethylbenzene (DMB), rhodamine B (RhB), methylene blue (MB), and methyl orange (MO) were bought from Dibaiin Shanghai. Other reagents are of analytical grade. Deionized water (>18 MΩ) was supplied from an Ultra-pure water system (Pall, USA).

X-ray powder diffractometer (XRD, MiniFlex600, Rigaku, Japan) was utilized for the analysis of TiO_2_, DA-TiO_2_, and DA-TiO_2_ (Ag) composites. Scanning electron microscopy (SEM, Hitachi E-1010, Japan) was utilized to characterize the as-prepared materials or coatings. The contact-angle measurement machine (Jinhe, Jiangsu, China) was applied for the hydrophobic analysis of the water on different coating surfaces. The light source for photocatalytic degradation was performed using a 450 W xenon lamp (XL-300, Shenyang). UV-3600 spectrophotometer with the holder for solid-phase measurements (Shimadzu, Japan) was used to monitor the absorbance values of different organic dye droplets deposited on the coatings for calculating the photocatalytic efficiencies of dyes.

### Preparation of TiO_2_ nanoparticles

An aliquot of 10 mL TBOT was mixed ultrasonically with 20 mL anhydrous ethanol in the water bath. Then, the TBOT solution was dropped into the solution containing 20 mL ethanol and 25 mL acetic acid at the dropping speed of 1 d s^−1^. After that, 0.70 g PEG was added by ultrasonically stirred at the adjusted pH 6.0. Then, the obtained light blue sol was put into the reaction kettle (100 mL) to be heated at 160 °C for 24 h. After being cooled down to room temperature, the resulting mixture was centrifuged and then washed for three times separately with water and ethanol. Finally, the products were dried at 60 °C for 12 h to be stored at 4 °C for the future usage.

### Preparation of super-hydrophobic DA-TiO_2_ (Ag)@PC formula

An aliquot of 10 mL DA (4.0 mg mL^−1^) in ethanol was mixed with 10 mL TiO_2_ with different concentrations in ethanol (0, 2.0, 4.0, 6.0, 8.0, 10.0, and 12.0 mg mL^−1^). Then, a certain amount of AgNO_3_ (final concentrations of 0, 1.0, 2.0, 3.0, 4.0, and 5.0 mM) was separately introduced to bestirred in dark for 24 h. The so obtained products were centrifuged and then washed for three times separately with water and ethanol. The resulting DA-TiO_2_ (Ag) composites containing about 4.5 wt% silver and about 23 wt% DA were dried at 60 °C for 24 h, and further stored in dark at 4 °C for the future usage. Subsequently, an aliquot of PC was first dissolved in DCM. Then, different quantities of DA-TiO_2_ (Ag) (0, 2.0, 4.0, 6.0, 8.0, 10.0, and 12.0 mg) were dissolved in 20 mL PC solution (10 wt %) to be stirred till they were dispersed well. The resulting formula mixture of DA-TiO_2_ (Ag)@PC were stored in DCM.

### Construction of super-hydrophobic E-DA-TiO_2_ (Ag)@PC coatings on various substrates

The clean substrates (i.e., glass, iron, ceramics, paper, wood, and textile) were first dipped separately into 20 mL glue solution for 30 s, then dipped into an aliquot of 20 mL DA-TiO_2_ (Ag)@PC in DCM at a certain dosage for 60 s, followed by the fast evaporation of the solvent. Furthermore, the substrates were dipped into DMB for 5 min to be etched, thus making the nanoporous E-DA-TiO_2_ (Ag)@PC coatings dried at room temperature. The so prepared super-hydrophobic surfaces were studied by SEM imaging and CA measurements. The substrate adhesion was necessarily investigated for the E-DA-TiO_2_(Ag)@PC coatings on the steel substrates by the Cross-cut tape test of ASTM D3359B-02 Method B^32^, with the results evaluated accordingly.

### Photocatalytic degradation of different dyes on E-DA-TiO_2_ (Ag)@PC coatings

An aliquot of 2.0 μL of different organic dyes of RhB, MB, and MO (1.0 × 10^−4^ M) in ethanol was separately dropped on the E-DA-TiO_2_ (Ag)@PC coatings deposited on various substrates under the xenon lamp light. Following that, the solid-phase UV-vis absorbance values of dye droplets were recorded every 10 min.

In addition, the experimental conditions for creating super-hydrophobic E-DA-TiO_2_ (Ag)@PC coatings were optimized mainly including different amounts of AgNO_3_ (0, 1.0, 2.0, 3.0, 4.0 and 5.0 mM) and DA- TiO_2_ (Ag) (0, 0.10, 0.20, 0.30, 0.40, 0.50 and 0.60 wt%). The solid-phase UV-vis absorbance values were recorded at time interval of 10 min for the droplets of RhB (originally 1.0 × 10^−4^ M) that were deposited on the coatings to be exposed under xenon lamp light. Thereafter, the photocatalytic degradation efficiencies (%) were calculated.

## Additional Information

**How to cite this article**: Li, Z. *et al*. Super-hydrophobic Silver-Doped TiO_2_ @ Polycarbonate Coatings Created on Various Material Substrates with Visible-Light Photocatalysis for Self-Cleaning Contaminant Degradation. *Sci. Rep.* **7**, 42932; doi: 10.1038/srep42932 (2017).

**Publisher's note:** Springer Nature remains neutral with regard to jurisdictional claims in published maps and institutional affiliations.

## References

[b1] Barthlott W. & Neinhuis C. Purity of the sacred lotus, or escape from contamination in biological surfaces. Planta 202, 1–8 (1997).

[b2] Feng L. . Super‐hydrophobic surfaces: from natural to artificial. Adv. Mater. 14, 1857–1860 (2002).

[b3] Boinovich L. B. & Emelyanenko A. M. Hydrophobic materials and coatings: principles of design, properties and applications. Russ. Chem. Rev. 77, 583–600 (2008).

[b4] Roach P., Shirtcliffe N. J. & Newton M. I. Progess in superhydrophobic surface development. Soft Matter. 4, 224–240 (2008). 10.1039/b712575p 32907233

[b5] Liu Y. . A electro-deposition process for fabrication of biomimetic super-hydrophobic surface and its corrosion resistance on magnesium alloy. Electrochim. Acta 125, 395–403 (2014).

[b6] Cheng Z. . From petal effect to lotus effect: a facile solution immersion process for the fabrication of super-hydrophobic surfaces with controlled adhesion. Nanoscale 5, 2776–2783 (2013). 23429404 10.1039/c3nr34256e

[b7] Kumar A. . Nano/Micro Engineered and Molecular Systems (NEMS), 2014 9th IEEE International Conference on, 2014.

[b8] Zhang X. . Polyelectrolyte multilayer as matrix for electrochemical deposition of gold clusters: toward super-hydrophobic surface. J. Am. Chem. Soc. 126, 3064–3065 (2004). 15012132 10.1021/ja0398722

[b9] Favia P. . Deposition of super-hydrophobic fluorocarbon coatings in modulated RF glow discharges. Surf. Coat. Technol. 169, 609–612 (2003).

[b10] Yamashita H. . Coating of TiO_2_ photocatalysts on super-hydrophobic porous teflon membrane by an ion assisted deposition method and their self-cleaning performance. Nucl. Instr. and Meth. B. 206, 898–901 (2003).

[b11] Li X. . Preparation of a super-hydrophobic poly (vinyl chloride) surface via solvent–nonsolvent coating. Polymer 47, 506–509 (2006).

[b12] Liu Y. . Artificial lotus leaf structures from assembling carbon nanotubes and their applications in hydrophobic textiles. J. Mater. Chem. 17, 1071–1078 (2007).

[b13] Sun C., Ge L.-Q. & Gu Z.-Z. Fabrication of super-hydrophobic film with dual-size roughness by silica sphere assembly. Thin Solid Films. 515, 4686–4690 (2007).

[b14] Zhang Y. . A rapid and efficient strategy for creating super-hydrophobic coatings on various material substrates. J. Mater. Chem. 18, 4442–4449 (2008).

[b15] Erbil, . Transformation of a simple plastic into a superhydrophobic surface. Science 299, 1377–1380 (2003). 12610300 10.1126/science.1078365

[b16] Wang L. & McCarthy T. J. Covalently Attached Liquids: Instant Omniphobic Surfaces with Unprecedented Repellency. Angew. Chem. Int. Ed. 55, 244–248 (2016). 10.1002/anie.201509385 26568536

[b17] Lin J. Y. . Fabrication of biomimetic superhydrophobic surfaces inspired by lotus leaf and silver ragwort leaf. Nanoscale 3, 1258–1262 (2011). 21270991 10.1039/c0nr00812e

[b18] Lu Y. . Robust self-cleaning surfaces that function when exposed to either air or oil. Science 347, 1132–1135 (2015). 25745169 10.1126/science.aaa0946

[b19] Li X. . Preparation of a super-hydrophobic poly(vinyl chloride) surface via solvent-nonsolvent coating. Polymer 47, 506–509 (2006).

[b20] Zhao N. . Fabrication of Biomimetic Superhydrophobic Coating with a Micro‐Nano‐Binary Structure. Macromol. Rapid Commun. 26, 1075–1080 (2005).

[b21] Chen X. . Increasing solar absorption for photocatalysis with black hydrogenated titanium dioxide nanocrystals. Science 331, 746–750 (2011). 21252313 10.1126/science.1200448

[b22] Yamanaka M. . Construction of superhydrophobic surfaces by fibrous aggregation of perfluoroalkyl chain-containing organogelators. Chem. Commun. 21, 2248–2250 (2006). 10.1039/b601485b 16718318

[b23] Schneider J. . Understanding TiO_2_ photocatalysis: mechanisms and materials. Chem. Rev. 114, 9919–9986 (2014). 25234429 10.1021/cr5001892

[b24] Liu G. . Photoassisted degradation of dye pollutants. 8. Irreversible degradation of alizarin red under visible light radiation in air-equilibrated aqueous TiO_2_ dispersions. Environ. Sci. Technol. 33, 2081–2087 (1999).

[b25] Fujishima A., Rao T. N. & Tryk D. A. Titanium dioxide photocatalysis. J. Photoch. Photobio. C 1, 1–21 (2000).

[b26] Dvoranova D., Brezova V., Mazúr M. & Malati M. A. Investigations of metal-doped titanium dioxide photocatalysts. Appl.Catal. B 37, 91–105 (2002).

[b27] He B.-L., Dong B. & Li H.-L. Preparation and electrochemical properties of Ag-modified TiO_2_ nanotube anode material for lithium–ion battery. Electrochem. Commun. 9, 425–430 (2007).

[b28] Yang D. . Synthesis of Ag/TiO_2_ nanotube heterojunction with improved visible-light photocatalytic performance inspired by bioadhesion. J. Phys. Chem. C 119, 5827–5835 (2015).

[b29] Yao W. . Synthesis and characterization of high efficiency and stable Ag_3_PO_4_/TiO_2_ visible light photocatalyst for the degradation of methylene blue and rhodamine B solutions. J. Mater. Chem. 22, 4050–4055 (2012).

[b30] Wu T. . Photoassisted degradation of dye pollutants. V. Self-photosensitized oxidative transformation of rhodamine B under visible light irradiation in aqueous TiO_2_ dispersions. J. Phys. Chem. B 102, 5845–5851 (1998).

[b31] Zhang L. . A facile approach to superhydrophobic coating from direct polymerization of “super glue”. Soft Matter. 7, 4050–4054 (2011).

[b32] American Society for Testing and Materials, ASTM, West Conshohocken, PA, D-3359-02 cross-cut tape test for adhesion.

[b33] Li R. . A highly specific and sensitive electroanalytical strategy for microRNAs based on amplified silver deposition by the synergic TiO_2_ photocatalysis and guanine photoreduction using charge-neutral probes. Chem. Commun. 51, 16131–16134 (2015). 10.1039/c5cc07277h 26391315

[b34] Rajh T. . Surface restructuring of nanoparticles: An efficient route for ligand-metal oxide crosstalk. J. Phys. Chem. B 106, 10543–10552 (2002).

